# Effects of α-Melanocortin Enantiomers on Acetaminophen-Induced Hepatotoxicity in CBA Mice

**DOI:** 10.3390/molecules14125017

**Published:** 2009-12-02

**Authors:** Petra Turčić, Mirna Bradamante, Karlo Houra, Nikola Štambuk, Tomislav Kelava, Paško Konjevoda, Saša Kazazić, Dražen Vikić-Topić, Biserka Pokrić

**Affiliations:** 1 Department of Pharmacology, Faculty of Pharmacy and Biochemistry, University of Zagreb, Domagojeva 2, 10000 Zagreb, Croatia; E-Mail: pturcic@pharma.hr (P.T.); 2 Department of Dermatology and Venerology, University Hospital Center Zagreb, Šalata 4, 10000 Zagreb, Croatia; E-Mail: mirna.bradamante@kbc-zagreb.hr (M.B.); 3 Department of Neurosurgery, University Hospital “Sestre Milosrdnice”, Vinogradska 29, 10000 Zagreb, Croatia; E-Mail: khoura@kbsm.hr (K.H.); 4 Ruđer Bošković Institute, Bijenička cesta 54, 10002 Zagreb, Croatia; E-Mails: pkonjev@irb.hr (P.K.); kazazic@irb.hr (S.K.); vikic@irb.hr (D.V-T.); pokric@irb.hr (B.P.); 5 Department of Physiology and Immunology, School of Medicine, University od Zagreb, Šalata 3, 10000 Zagreb, Croatia; E-mail: tkelava@mef.hr (T.K.)

**Keywords:** enantiomer, α-MSH, hepatoprotection, hepatotoxicity, antibody, CD spectroscopy

## Abstract

Proteins and peptides in mammals are based exclusively on l-amino acids. Recent investigations show that d-amino acids exhibit physiological effects *in vivo*, despite of their very small quantities. We have investigated the hepatoprotective effects of the l- and d-enantiomers of α-melanocortin peptide (α-MSH). The results showed that *peptide-enantiomerism* is related to the protective effects of melanocortin peptides *in vivo*. l-α-MSH exhibited potent hepatoprotective effect in the experimental model of acetaminophen induced hepatotoxicity in male CBA mice, while its d-*mirror image* was inefficient. Furthermore, the antibody to the l-peptide did not recognize the d-structure. The results indicate that the opposite peptide configuration may be used to modulate its function and metabolism *in vivo* and *in vitro*.

## 1. Introduction

**Figure 1:**
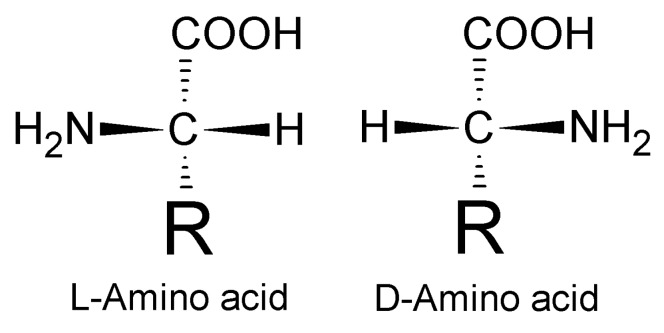
Representation of l- and d-amino acid structure.

It has been long believed that only the l-enantiomers of amino acids are present in higher animals [1,2,3,4,5]. Recent advances in analytical chemistry and progress in peptide synthesis have however enabled the investigation of amino acid and peptide d-enantiomers (Figure 1), both from the biochemical and physiological standpoint [1,3]. Intermolecular interactions of polypeptides or antibodies to their receptors are known to be sterically constrained [6,7,8,9]. However, little is known on “how the influence of inversion of configuration of all stereogenic centers in a protein influences on its function?” [2]. Consequently, we performed a comparative investigation of α-melanocortin (α-MSH) l- and d-enantiomers with respect to their structure, *in vitro* binding, and *in vivo* protective effects. We have investigated α-MSH peptide because its l-enantiomer is an ancient and evolutionary conserved tridecapeptide hormone that exhibits well defined protective and antiinflammatory effects on different tissues and organs, including liver [10,11,12]. α-MSH derives from post-translational processing of the pro-opiomelanocortin (POMC) propeptide [10,11,12].

## 2. Results and Discussion

### 2.1. Circular Dichroism Spectroscopy of α-MSH Enantiomers 

**Figure 2:**
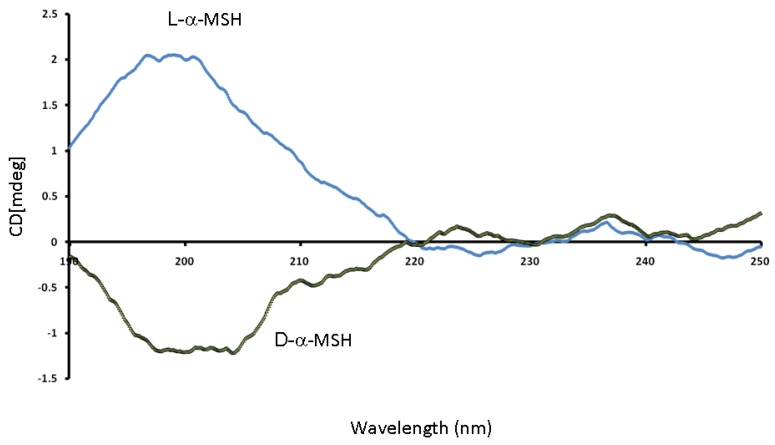
CD spectra of l-α-MSH and d-α-MSH enantiomers (10 mM, pH 7.4, sodium phosphate buffer at peptide concentration of 0.1 mM).

The circular dichroism (CD) spectra of l-α-MSH and d-α-MSH enantiomers in Figure 2 show an opposite sign of ellipticity degree caused by differential absorption of circular polarized light components proving their opposite chiral activity [13]. According to the spectral profile both peptide isomers in aqueous solution of sodium phosphate buffer (10 mM, pH 7.4) exhibit predominantly random coil structure form [14,15], having maximum ellipticity around 200 nm arising out of the π-π transition of the peptide amide bond (Figure 2).

### 2.2. Competitive Enzymatic Immunoassay (EIA) of α-MSH Enantiomers

**Figure 3:**
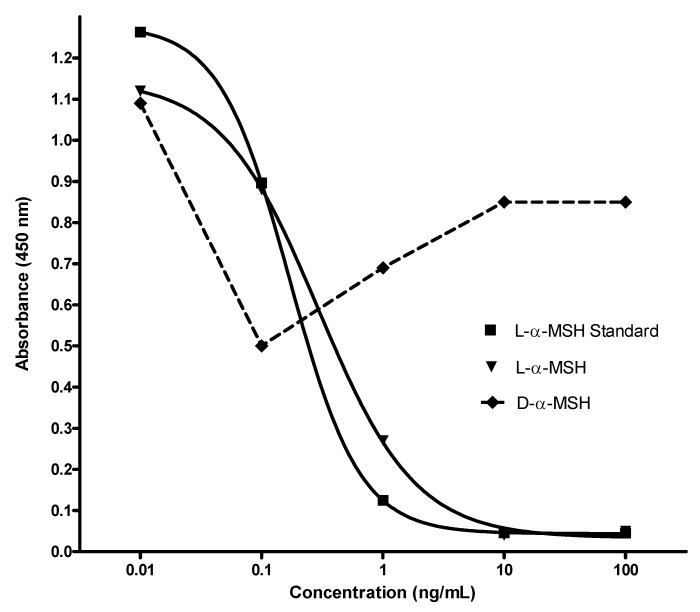
Detection of l- and d-α-MSH by means of competitive enzymatic immunoassay.

l-α-MSH and d-α-MSH enantiomers were quantified by means of the competitive enzymatic immunoassay kit (Phoenix Pharmaceuticals Inc., USA) [16,17]. Results presented in Figure 3 show matching curve paterns of l-α-MSH EIA standard and tested l-α-MSH enantiomer. d-α-MSH enantiomer was not recognized by the competitive EIA procedure, *i.e.*, by the primary antibody to l-α-MSH peptide (Figure 3).

Our results are in line with several other reports indicating that opposite peptide configurations of enantiomers may be used to modulate their function, metabolism and antibody binding *in vitro* [6,7,8,9]. They support the findings of Sela and Zisman [18] that peptides consisting of d-amino acids exhibit stereospecific antibody responses. Benkirane *et al*. [8] and Guichard *et al*. [9] also described that antibody response in mice is stereospecific and subclass dependant.

It is currently unknown if d-α-MSH enantiomer or its small fragments bind to melanocortin receptors. In mammalian tissues d-amino acids are first converted into the corresponding α-keto acids by d-amino-acid oxidase (DAO) [3,19]. During this process potentially toxic ammonia (NH_3_) and hydrogen peroxide (H_2_O_2_) are produced [3]. In the second step α-keto acids are stereospecifically converted to their corresponding l-amino acids by transaminases [19]. Consequently, the process of D→L amino acid conversion may influence hepatocyte metabolism and function. 

### 2.3. Treatment of Hepatotoxicity with α-MSH Enantiomers

Acetaminophen (APAP) produces liver lesions in two phases - *intrinsic* and *extrinsic*. In the *intrinsic* phase the reactive metabolite *N*-acetyl-*p*-benzoquinone imine (NAPQI) covalently binds to important intracellular proteins which leads to damage of centrilobular regions of liver [20,21]. In the *extrinsic* phase the release of intracellular content is accompained with liberation of numerous cytokine and chemokine mediators from nonparenchymal and nonhepatic cell types [20]. The final result is the strong inflammatory response of the liver, and the model is a well known screening model for hepatoprotective drugs [20,21]. The effect of tested substances is estimated by plasma alanine aminotransferase (ALT) and aspartate aminotransferase (AST) activity measurement and histopathological grading of liver lesions [20,21]. 

l-α-MSH enantiomer exhibited hepatoprotective dose dependent effects (Table 1, Table 2 and Table 3). ALT and AST were significantly lower in the group trested with 2.5 mg/kg of l-α-MSH with respect to the control group treated with 0.9% NaCl (Table 1 and Table 2). d-α-MSH enantiomer did not show any protective effects. Biochemical findings of l-α-MSH hepatoprotection and d-α-MSH inefficiency were confirmed by the histopatological grading of the lesions (Table 3). The results show that only naturally occurring l-α-MSH peptide has protective effects on liver damage. This is in line with the protective effects of l-α-MSH reported for thioacetamide-, carbon tetrachloride- and endotoxin-induced liver lesions [22,23,24].

**Figure 4:**
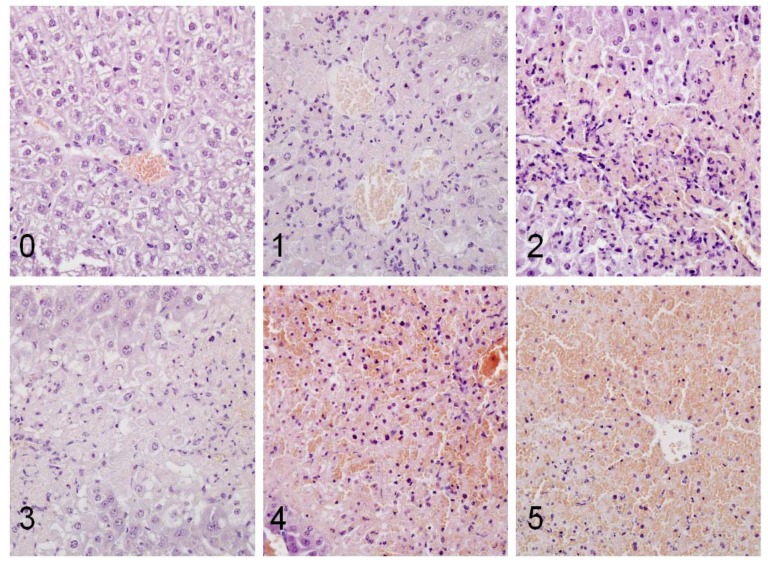
Histopathological analysis of liver sections using a light microscopy (hematoxilin and eosin staining, magnification ×200). Presence and intensity of lesions was graded on a scale from 0 to 5. 0 = no lesions ; 1 = minimal lesions, isolated necrotic cells; 2 = mild lesions, from 10% to 25% of necrotic cells or mild diffuse degenerative changes; 3 = moderate lesions, from 25 to 40% of necrotic cells; 4 = marked lesions, from 40 to 50% of necrotic cells; 5 = severe lesions, more than 50% of necrotic cells.

The histological changes seen in acetaminophen poisoning are centrilobular hepatic necrosis and sinusoidal congestion. In severe cases with scores 3-5 (Figure 4, Table 3) submassive (bridging) or panacinar (massive) necrosis is seen [25,26]. This pattern of necrosis reflects the role of P450 isozyme for bioactivation (CYP2E1), which is concentrated in this part of the hepatic acinus, as well as the fact that glutathione levels are lower in centrilobular zone [25,26]. Inflammation is not a significant feature of severe lesions because the necrosis unables quantification of the infiltrate [26]. Inflammatory infiltrate is seen in the groups with mild lesions and lack of necrosis, *i.e.*, in groups with scores 1-2 (Figure 4, Table 3). l-α-MSH exerts its antiinflammatory effects by activating melanocortin receptors, leading to adenyl cyclase activation and subsequent increase of intracellular cAMP concentration [11,12]. This prevents activation of nuclear factor κB, and subsequently leads to reduction of pro-inflammatory mediators synthesis and adhesion molecules expression [11,12].

## 3. Experimental

### 3.1. Circular Dichroism Experiment

The circular dichroism spectra were acquired at room temperature with a Jasco J-810 CD spectropolarimeter using quartz precision cells of 0.2 mm path length. The following scan parameters were used: high sensitivity (5 mdeg), 1-nm bandwidth, 1-s response time, 0.2-nm step resolution and 50 nm/min. scan speed. Spectrum was an average of five continuous scans, measured between 190 and 250 nm. l-α-MSH and d-α-MSH isomer samples (>95% purity; GenScript, USA) were prepared for the analysis by stock solution 1:50 dilution into sodium phosphate buffer (10 mM, pH 7.4), to give a final peptide concentration of 0.1 mM. The acquired spectra were corrected by subtracting blank runs on peptide-free sodium phosphate buffer using JASCO proprietary software Spectra Analysis version 1.53.04 and presented as the ellipticity in degrees without normalization. 

### 3.2. Quantification of α-MSH Enantiomers by Means of Competitive EIA 

l-α-MSH and d-α-MSH enantiomers were quantified by means of a commercially available competitive enzymatic immunoassay kit (Phoenix Pharmaceuticals Inc., USA) [16,17]. First, l-α-MSH standard, l-α-MSH and d-α-MSH were added to microtiter plate wells pre-coated with secondary antibody. Primary antibody and then biotinylated l-α-MSH were added. After 2-hour incubation supernatants were discarded and wells were washed. Then, solution of horseradish peroxidase bound to streptavidin (SA-HRP) was added, system was incubated 1 hour and washed further. The 3,3′,5,5′-tetramethylbenzidine (TMB) substrate solution was added in dray wells and incubated for 1 hour. By addition of 2 N HCl blue substrate colour was changed to yellow and the absorbance (optical density) was read at 450 nm. α-MSH concentrations were expressed as ng/mL (minimum detectable concentration = 0.14 ng/mL, as indicated by the manufacturer) [16].

### 3.3. Animals

Experimental animals were male CBA mice, 12–16 weeks old, weighing 20–25 g and bred at the Ruđer Bošković Institute. The animals were kept in a room with constant temperature (22 ± 1 °C) and dark-light cycle (12h/12h). Mice were food-fasted with free access to water 24 hours prior to inducing liver damage by acetaminophen. Four hours after the substance administration they were fed by standard laboratory food and given water *ad libitum*. 

### 3.4. Treatment Regimen (Hepatotoxicity Model)

Hepatotoxicity was induced following the procedure described by Guarner *et al*. [27], with slight modifications [28]. To induce hepatic drug-metabolizing enzymes mice were given phenobarbitone-sodium (Kemika, Zagreb, Croatia) in their drinking water for 7 days in a dose of 0.3 g/L. Thereafter, mice were fasted overnight and acetaminophen (Krka, Novo Mesto, Slovenia) 150 mg/kg was given intragastrically (i.g.), via a gastric tube, in a volume of 0.5 mL. Mice were re-fed after 4 hours. All tested substances were given intraperitoneally (i.p.) 1 hour before acetaminophen administration, in a volume of 0.2 mL. Control animals were treated with saline (0.9% NaCl). The size of experimental groups was 6–8. Mice that spontaneously died were excluded from histopathological or biochemical analysis.

### 3.5. Test Compounds

l- and d-enantiomers of α-MSH (Ac-SYSMEHFRWGKPV-NH_2_, >95% purity; GenScript, USA), were administered intraperitonealy (i.p.) in four progressive doses (0.1 mg/kg, 0.5 mg/kg, 1.0 mg/kg and 2.5 mg/kg) dissolved in 0.9% NaCl 1 hour prior to the administration of APAP.

### 3.6. Histopathological Estimation of Liver Damage

Sections of the liver were fixed in 10% phosphate buffered formalin, embedded in paraffin, sectioned at 4 µm, and stained with hematoxilin and eosin. Sections were examined by using light microscope at ×100 magnification. Grading of the liver lesions from 0-5, proposed by Silva *et al*. [29] was applied: 0 = no lesions detected; 1 = minimal lesions, isolated necrotic cells; 2 = mild lesions, from 10% to 25% of necrotic cells or mild diffuse degenerative changes; 3 = moderate lesions, from 25 to 40% of necrotic cells; 4 = marked lesions, from 40 to 50% of necrotic cells; 5 = severe lesions, more than 50% of necrotic cells. Lesions of 3 and more points were considered to be a significant liver damage.

### 3.7. Plasma Transaminase Activity

Mice were sacrificed 24 hours after acetaminophen application. Alanine aminotransferase (ALT) and aspartate aminotransferase (AST) activity was determined from plasma by standard laboratory techniques. Plasma was separated by centrifugation for 5 min at 8,000 g, and was stored at −20 °C for 24 h before transaminase activity determination.

### 3.8. Data Analysis

Statistical analysis was performed with KyPlot Software (version 4.0). Kruskall-Wallis test and Steel’s test (P, Table 1, Table 2 and Table 3) were used to test the differences between effects of applied neuropeptide doses and control group (0.9% NaCl). All applied tests were two-tailed. P ≤ 0.05 were considered as statistically significant.

## 4. Conclusions

(1). Both enantomers of α-MSH peptide, when diluted 1:50 in sodium phosphate buffer (10 mM, pH 7.4), exhibit predominantly random coil structure.
(2). Antibody to l-α-MSH did not recognize d-α-MSH enantiomer.
(3). l-α-MSH enantiomer was hepatoprotective in the experimental model of acetaminophen induced hepatotoxicity in male CBA male mice, while d-α-MSH was inefficient.
(4). Peptide-enantiomerism should be considered in the investigations of peptide bio- and immuno-reactivity.

## Figures and Tables

**Table 1:** Aspartate aminotransferase activity (U/L) in plasma of the control and α-MSH treated animals 24 h after acetaminophen administration.

Substance	i.p. dose	Mean	SD	Median	P value
Control	0.9% NaCl	6767.25	6468.60	5434.0	
l-α-MSH	0.10 mg/kg	4880.43	3891.38	3691.0	>0.999
l-α-MSH	0.50 mg/kg	2613.88	1114.85	2368.5	>0.999
l-α-MSH	1.00 mg/kg	1418.50	1228.44	905.5	0.587
l-α-MSH	2.50 mg/kg	539.50	459.62	414.0	0.031
d-α-MSH	0.10 mg/kg	1756.21	1031.83	1940.0	0.996
d-α-MSH	0.50 mg/kg	3774.53	2082.77	3460.0	>0.999
d-α-MSH	1.00 mg/kg	3082.32	2810.30	3730.0	0.806
d-α-MSH	2.50 mg/kg	2754.30	1388.61	3400.0	0.999

**Table 2:** Alanine aminotransferase activity (U/L) in plasma of the control and α-MSH treated animals 24 h after acetaminophen administration.

Substance	i.p. dose	Mean	SD	Median	P value
Control	0.9% NaCl	9550.00	9213.15	7321.5	
l-α-MSH	0.10 mg/kg	8983.67	4654.38	7901.0	0.998
l-α-MSH	0.50 mg/kg	4292.00	1422.14	3945.0	>0.999
l-α-MSH	1.00 mg/kg	2671.25	2235.06	2167.5	0.589
l-α-MSH	2.50 mg/kg	585.75	1424.17	89.0	0.023
d-α-MSH	0.10 mg/kg	3602.50	2068.51	3820.0	0.996
d-α-MSH	0.50 mg/kg	5752.22	1847.53	5630.0	>0.999
d-α-MSH	1.00 mg/kg	5162.47	4226.78	6480.0	0.808
d-α-MSH	2.50 mg/kg	4932.86	2238.02	5580.0	>0.999

**Table 3:** Intensity of liver lesions 24 h after acetaminophen administration in controls and α-MSH treated animals.

Substance	i.p. dose	Mean	SD	Median	P value
Control	0.9% NaCl	4.00	1.20	4.5	
l-α-MSH	0.10 mg/kg	4.17	0.98	4.5	0.999
l-α-MSH	0.50 mg/kg	3.33	0.52	3.0	0.560
l-α-MSH	1.00 mg/kg	2.29	0.49	2.0	0.032
l-α-MSH	2.50 mg/kg	2.00	0.76	2.0	0.018
d-α-MSH	0.10 mg/kg	4.40	0.89	5.0	0.997
d-α-MSH	0.50 mg/kg	4.60	0.55	5.0	0.971
d-α-MSH	1.00 mg/kg	4.20	1.10	5.0	>0.999
d-α-MSH	2.50 mg/kg	4.89	0.38	5.0	0.506
